# Supplementation of Superfine Powder Prepared from *Chaenomeles speciosa* Fruit Increases Endurance Capacity in Rats via Antioxidant and Nrf2/ARE Signaling Pathway

**DOI:** 10.1155/2014/976438

**Published:** 2014-12-24

**Authors:** Ka Chen, Jia You, Yong Tang, Yong Zhou, Peng Liu, Dan Zou, Qicheng Zhou, Ting Zhang, Jundong Zhu, Mantian Mi

**Affiliations:** Research Center for Nutrition and Food Safety, Institute of Military Preventive Medicine, Third Military Medical University, Shapingba District, Chongqing 400038, China

## Abstract

*Chaenomeles speciosa* fruit is a traditional herb medicine widely used in China. In this study, superfine powder of *C. speciosa* fruit (SCE), ground by supersonic nitrogen airflow at −140°C, was investigated to assess its *in vitro* antioxidant activity and *in vivo* antiphysical fatigue activity. SCE was homogenous (*d* < 10 *μ*m) and rich in antioxidants like polyphenols, saponins, oleanolic acid, ursolic acid, ascorbic acid, and SOD. According to the *in vitro* experiments, SCE displayed promising antioxidant activity with powerful FARP, SC-DPPH, and SC-SAR activities. According to the *in vivo* experiments, rats supplemented with SCE had prolonged exhaustive swimming time (57%) compared to the nonsupplemented rats. Meanwhile, compared to the nonsupplemented rats, the SCE-supplemented rats had higher levels of blood glucose and liver and muscular glycogen and lower levels of LA and BUN. Lower MDA, higher antioxidant enzymes (SOD, CAT, and GSH-Px) activities, and upregulated Nrf2/ARE mediated antioxidant enzymes (HO-1, Trx, GCLM, and GCLC) expression were also detected in the supplemented group. This study indicates that SCE is a potent antioxidant and antifatigue agent, and SCE could be a promising raw material for the food and pharmaceutical industries.

## 1. Introduction

Exercise-induced fatigue has been extensively studied in athletes, military, and industry personnel. Exercise intolerance, characterized by difficulty in sustaining voluntary activities, is the well-known consequence of this physical fatigue. Although the underlying mechanism of exercise-induced fatigue has not been fully clarified, reactive oxygen species (ROS), especially those derived from skeletal muscle, have been regarded as the crucial factor in fatigue development and progress [1]. In recent studies, several natural antioxidants, such as green tea extract [2, 3], ginsengs [4], red mold rice [5], and maca extract [6], have been proved to be effective in improving physical endurance. So, people pay more attention to seek natural compounds with potent antioxidant activity to improve physical performance, postpone fatigue, and accelerate the elimination of fatigue in human beings.

*Chaenomeles speciosa* (Sweet)* Nakai* (*Rosaceae*) is a traditional Chinese herb widely used in the treatment of dyspepsia and several inflammatory diseases. Recent studies have reported its immunomodulatory effect, hepatoprotective effect, antinociceptive effect, antitumor effect, and antihyperlipidemic effect [7–10]. As a result of its various health effects revealed,* C. speciosa* has gained considerable attention both in Chinese medicine and in food industry. Studies have reported that polyphenols (e.g., phenolics, flavonoids, quercetin, etc.), alkaloids, organic acids, glycosides, and some amino acid derivatives are the main bioactive components of* C. speciosa* fruits [7–10]. Among these ingredients, polyphenols, saponins, oleanolic acid (OA), ursolic acid (UA), ascorbic acid, and superoxide dismutase (SOD), which are powerful antioxidants [7, 10], might have antifatigue properties. However, there are no published reports on the antifatigue property of* C. speciosa* produced in China.

In the present study, we identified whether SCE could produce positive effects on endurance capacity. To maintain the important bioactive components and avoid the incorporation of additional chemical agents, a modified superfine grinding process, which included flash evaporation and superfine grinding using a 3.08 Mach supersonic airflow at −140°C, was used in the preparation of SCE. In this work, quality characters of SCE such as particle size, the main antioxidants contents, and* in vitro* antioxidant activities were firstly analyzed. Then, the* in vivo* antifatigue activity was assessed using the weighted forced swim test (WFST) with rats feeding with SCE for 14 days, and biomarkers related to fatigue and oxidative stress were measured, and the nuclear factor erythroid 2-related factor 2 (Nrf2)/antioxidant response element (ARE) signaling pathway in skeletal muscle was further detected. This work would provide an important basis for developing SCE as a novel antifatigue agent and facilitate further development and utilization of* C. speciosa*.

## 2. Materials and Methods

### 2.1. Chemicals

Gallic acid, ellagic acid, chlorogenic acid, caffeic acid, catechin, quercetin, thiobarbituric acid, DPPH, 2, 4, 6-tripyridyl-s-triazine (TPTZ), and ferrozine were purchased from Sigma Chemical Co. (St. Louis, MO, USA). OA and UA standard were obtained from Siyi Biotechnology Company (Chengdu, China). Reagents for measuring SC-SAR, TAOC, thiobarbituric acid reacting substances (TBARS), total GSH, SOD, CAT, GSH-Px, and BUN were bought from Nanjing Jiancheng Institute of Biology and Engineering (Nanjing, China). cDNA synthesis kit and real-time PCR were purchased from Bio-Rad (Hercules, CA). Primers for HO-1, Trx, GCLM, GCLC, and *β*-actin were purchased from Invitrogen (Shanghai, China). Primary antibody against Nrf2 and Lamin B were obtained from Abcam (Cambridge, MA) and other antibodies for detection of HO-1, Trx, GCLC, GCLM, and*β*-actin were from Novus Biologics (Littleton, CO). All the organic solvents and chemicals used in this study were of analytical grade.

### 2.2. SCE Preparation

*C. speciosa* fruit was collected from the Qijiang district of the city of Chongqing in China during the early month of September (2012) and was stored at −20°C until used. The* C. speciosa* fruit was washed, enucleated, and ground into coarse powder (*d* < 30 mesh). Then, the coarse powder was subjected to an automatic flash dryer. When the water content of coarse powder was <8%, the powder was then ground to achieve a superfine powder consistent in an UF-250 airflow micronizer (Saishan Powder Machinery Manufacturing Co. Ltd., Shanghai, China) with a 3.08 Mach supersonic nitrogen airflow at −140°C. Meanwhile, the traditional herbal pieces for decoction of* C. speciosa* fruit (TCE) were processed according to the Chinese pharmacopoeia (2010 version). TCE, which served as the control, was analyzed for appraising the antioxidant quality indexes of SCE.

### 2.3. Morphological Character and Particle Size Distribution Analysis of SCE

Morphological characterization of SCE particles was performed on images acquired using a scanning electron microscope (SEM). The particle size distribution of SCE was measured by a laser diffraction instrument (Mastersizer 2000, UK).

### 2.4. The Main Antioxidants Contents of SCE Analysis

HPLC analysis of OA, UA, and the representative phenolics including gallic acid, ellagic acid, chlorogenic acid, caffeic acid, catechin, and quercetin was carried out on an Alliance HPLC system (Waters, USA; see Materials and Methods in Supplementary Material available online at http://dx.doi.org/10.1155/2014/976438). TPC was measured by Folin-Ciocalteu assay and total saponins were detected using spectrophotometry method as previously described [7]. Ascorbic acid detection was followed by 2,6,-dichloro-phenol-indophenol titrimetric analysis and the activity of SOD detection used the total SOD assay kit with WST-1 [7].

### 2.5. Assessment of the* In Vitro* Antioxidant Activity of SCE

The SC-DPPH, FRAP assays were measured according to the methods reported by us [7]. And the SC-SAR assay was detected using a commercial kit.

### 2.6. Assessment of the* In Vivo* SCE Antioxidant and Antifatigue Activities

#### 2.6.1. Animals and Experimental Design

Forty adult Sprague-Dawley rats (weight: 210 ± 10 g) were obtained from the Experimental Animal Center of the Third Military Medical University. After one week on an AIN-93 diet, rats were randomly divided into two groups: control group (Con.) and SCE group (SCE). The SCE group and Con. group, respectively, received 1.0 g/kg body weight of SCE in 10% PBS solution (intragastric gavage (ig)) or 10% PBS for 17 days at 9:00 am. From the 1st to the 13th day, each group underwent a swimming test for 5 min without any weights; the swimming test was performed between 1:00 and 3:00 pm. On the 14th day, each group underwent a WFST. According to the results obtained from the WFST, the rats were further divided into four groups: nonexercise (NEx) or control group, nonexercise with SCE supplementation group (NEx + SCE), exercise group (Ex), and exercise with SCE supplementation group (Ex + SCE). On the last three days, the Ex and Ex + SCE groups underwent an exhaustive exercise every day (i.e., WFST for 20 min, twice each day) to induce physical fatigue. The other two groups were allowed to rest. Following the 18th day, the animals were sacrificed and blood samples, liver, and gastrocnemius muscle were quickly obtained. All animal procedures were performed according to the Third Military Medical University Institutional Animal Care and Use Committee approved protocols.

#### 2.6.2. WFST

During 7 days, rats underwent a swimming test for 5 min without any weights. The WFST was subsequently performed in a round tank (100 × 50 × 50 cm) maintained at 33°C with a 75 cm water depth. The rats performed the WFST until they reached exhaustion. The weight, which was attached to their tails, was approximately 5% of their body weights. It was assumed that the rats had reached exhaustion when they failed to rise to the surface to breathe within a period of 10 s.

#### 2.6.3. Measuring Biochemical Parameters Related to Fatigue and Redox System

The levels of glucose, glycogen, LA, BUN, MDA, SOD, TAOC, CAT, and GSH-Px were measured using commercial kits.

#### 2.6.4. Real-Time PCR and Western Blot Analyses

Total RNA (1 *μ*g) from the gastrocnemius muscle was used to perform real-time PCR with the SYBR Green PCR Master Mix kit. Primers for HO-1, Trx, GCLM, GCLC, and *β*-actin were purchased from Invitrogen (Shanghai, China; see Table 1). The nuclear or total protein of gastrocnemius muscle was blotted with the primary antibodies against Nrf2, Lamin B, GSH, Trx, GCLC, GCLM, and *β*-actin. The band optical density was determined by Quantity One software (Bio-Rad Lab, Hercules, CA, USA).

### 2.7. Statistical Analyses

The experimental data are expressed as mean ± standard error and analyzed with the SPSS 17.0 software. The exhaustive swimming time data were analyzed by an unpaired sample *t*-test. The other results were analyzed by two-way ANOVA and Tukey's post hoc test. The significance level was set at *P* < 0.05.

## 3. Results and Discussion

### 3.1. Powder Yield and Particle Size of the Prepared SCE

**Figure 1:**
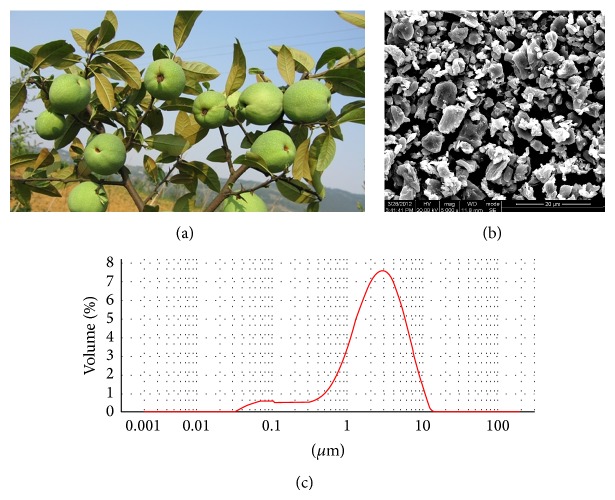
SEM images of the superfine powder from* C. speciosa* fruit (SCE) and its particles distribution diagram. (a) Fresh fruit of* C. speciosa*; (b) representative SEM images of SCE; (c) particles distribution diagram of SCE.

*C. speciosa* is a traditional herb that has been cultivated in China for thousands of years (Figure 1(a)). The traditional use of* C. speciosa* fruit, such as the TCE, cannot fulfill the variety demand. Although several technologies including extractions with enzymes and ethanol have been used in the deep processing of* C. speciosa*, bioactive components loss and chemical agents incorporation were big problems that we were confronted with [7, 10]. Nowadays, superfine grinding technology has begun to be used in foods processing, which would lead to better dispersibility, higher nutrient solubility, and bioavailability [11–14]. In this study, a modified superfine grinding technology was used to prepare SCE. Fresh fruit (14.0 kg) was processed to 1.82 kg powder and the yield of SCE was about 13%. During the SCE preparation, no chemicals were additionally added thereby ensuring its safety and purity. The morphology after milling changed considerably (Figure 1(b)), and the particle size of SCE was mainly distributed in 1–10 *μ*m (Figure 1(c)). A particle size of 0.5–10 *μ*m (in diameter) can significantly increase nutrient leaching, and superfine powder is easily incorporated into food matrices [13, 14]. Therefore, SCE may contribute to higher nutrient absorption and is suitable in the development of convenient food and pharmaceutical products.

### 3.2. Antioxidants Contents of the Prepared SCE

Previous studies have reported that the presence of oxygen and heat increases polyphenol and other antioxidants degradation. Some studies have reported that polyphenols such as EGCG, EGC, and EC are considerably reduced during superfine grinding [14]. In this study, to reduce the ruin effects of oxygen and heat on antioxidants, a coarse powder of* C. speciosa* fruit underwent superfine grinding by a 3.08 Mach supersonic nitrogen airflow at <−140°C; in these conditions, oxygen exposure and high temperatures were prevented. Phytochemical investigations on* C. speciosa* fruit reported that many classes of chemical groups contributed to its varieties of pharmacological function [7–10]. As shown in Table 2, superfine grinding significantly increased OA and UA, total polyphenols, gallic acid, ellagic acid, chlorogenic acid, caffeic acid, catechin, and quercetin concentrations when compared to TCE. To further determine the* in vitro* antioxidant activity of SCE, radical scavenging and reducing activity tests were performed. As shown in Table 2, SCE had higher DPPH IC_50_ value, SC-SAR activity, and FRAP activity than that of TCE. Above all, the development of the modified superfine grinding yielded a final product which is homogeneous and rich in antioxidants with powerful antioxidant activities.

### 3.3. SCE Prolonged the Exhaustive Swimming Time

In our preliminary experiments, a range of doses (0.2, 1.0, and 5.0 g/kg body weight/day) of SCE were studied to determine the most effective dose. Unfortunately, during the supplementation period, the 5.0 g/kg SCE dose resulted in loss of appetite and a reduction in body weight. Studies have suggested that polyphenols have strong effects on body tissues (e.g., severe chemical burns); thus the above symptoms might have been attributed to the high polyphenol content of SCE [7]. With the other two SCE doses, however, there were no obvious side effects. Based on the preliminary experiments (data not shown), the chosen SCE dose was 1.0 g/kg body weight/day.

**Figure 2:**
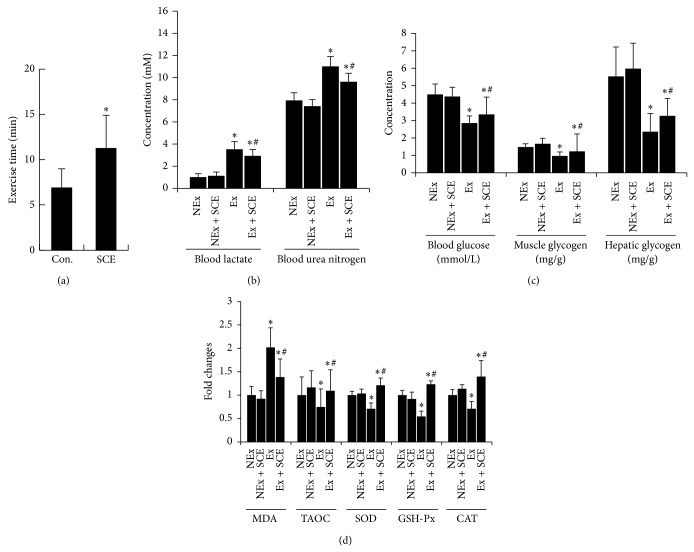
Antifatigue effect of the superfine powder prepared from* C. speciosa* fruit (SCE). (a) Exhaustive swimming time of rats in WFST test. Values represent mean ± SE. ^*^ *P* < 0.05 versus Con.: control group; SCE: SCE-supplemented group. (b) Blood lactic acid and blood urea nitrogen of rats exposed to exhaustive exercise. (c) Blood glucose, muscle glycogen, and liver glycogen of rats exposed to exhaustive exercise. (d) Oxidative-antioxidative statue of rats exposed to exhaustive exercise. Fold change was equal to that of the corresponding NEx group. Values represent mean ± SE. ^*^ *P* < 0.05 versus NEx group; ^#^ *P* < 0.05 versus Ex group. NEx: nonexercise group; NEx + SCE: nonexercise with SCE supplementation group; Ex: exercise group; Ex + SCE: exercise with SCE supplementation group.

A direct measure of antifatigue effect is the increase in exercise tolerance. WFST is a classical exercise model to evaluate antifatigue; it works well for evaluating the endurance of mice/rats and is highly reproducible [15–18]. Reduced susceptibility to fatigue is correlated with longer exhaustive swimming times. In this study, SCE significantly prolonged exhaustive swimming time from approximately 7 min to 11 min (Figure 2(a)). The results indicate that SCE has antifatigue activity and could increase endurance.

### 3.4. Effects of SCE on Biomarkers Related to Fatigue

Blood glucose homeostasis plays an important role in increasing endurance. Glycogen, an important energy source during exercise, maintains blood glucose within a physiological range. The amount of glycogen stored in the body reflects the speed and degree to which fatigue will occur [15–18]. As shown in Figure 2(b), SCE inhibited the exhaustive exercise-induced reduction of the blood glucose and the muscle and liver glycogen levels (*P* < 0.05). Next, we detected the sensitive fatigue indicators including LA and BUN, which were metabolism products of carbohydrate and protein, respectively [15–18]. Results showed that SCE reduced the exhaustive exercise-induced elevation of serum LA and BUN (Figure 2(c), *P* < 0.05). These results suggest that the blood glucose-homeostatic ability and reduced accumulated by-products of metabolism may be related to an improvement in exercise metabolism and antifatigue activity of SCE.

### 3.5. SCE Enhanced Antioxidative Enzymes in Rats That Underwent Exhaustive Exercise

In this study, we measured serum markers of oxidative stress including MDA, TAOC, SOD, GSH-Px, and CAT. MDA is one of the end-products of lipid peroxidation process; its levels increase during strenuous exercises [10, 19]. As shown in Figure 2(d), after three days of exhaustive exercise, the MDA level of the Ex + SCE group was lower than that of the Ex group (*P* < 0.05). TAOC was measured to assess the serum total antioxidant status. Serum antioxidant enzymes including SOD, GSH-Px, and CAT are important for scavenging free radicals and for maintaining normal cellular physiology [15, 20, 21]. In the present study, SCE inhibited the exhaustive exercise reduction of serum TAOC and activities of SOD, GSH-Px, and CAT (Figure 2(d)). These results provide evidence that SCE supplementation attenuates the exercise-induced oxidative stress and helps restore the oxidant-antioxidant balance.

### 3.6. SCE Regulated Nrf2/ARE Pathway in Rats That Underwent Exhaustive Exercise

**Figure 3:**
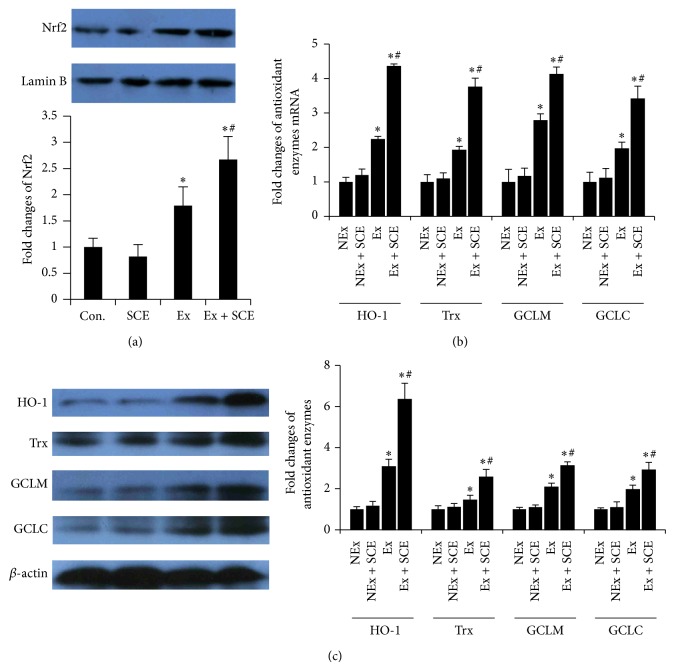
Effect of the superfine powder prepared from* C. speciosa* fruit (SCE) on Nrf2/ARE signal pathway of rats exposed to exhaustive exercise. (a) Nrf2 protein levels in gastrocnemius muscle. (b) mRNA levels of ARE related antioxidant enzymes in gastrocnemius muscle. (c) Protein levels of ARE related antioxidant enzymes in gastrocnemius muscle. Fold changes were quantified as the target protein or mRNA levels equal to the corresponding internal control (Lamin B or*β*-actin) in the NEx group. Values represent mean ± SE. ^*^ *P* < 0.05 versus NEx group; ^#^ *P* < 0.05 versus Ex group. NEx: nonexercise group; NEx + SCE: nonexercise with SCE supplementation group; Ex: exercise group; Ex + SCE: exercise with SCE supplementation group.

Nrf2 was reported to play a key role in regulating oxidative stress [22, 23]. Under basal conditions, Nrf2 is sequestered in the cytoplasm by Keap1 and rapidly degraded in an ubiquitin-proteasome-dependent manner, whereas under conditions of oxidative stress, Nrf2 escapes Keap1-mediated repression and translocates into nucleus to activate the expression of antioxidant and phase II drug-metabolizing enzymes through ARE. Recent studies have revealed that Nrf2-dependent modulation of redox system was the alternative mechanisms of flavones (quercetin and curcumin) cell protection, which is beyond its long-established ROS scavenging properties [22, 23]. In this study, although increased nuclear location of Nrf2 was observed (Figure 3(a)), the MDA accumulation displayed exhaustive exercise-induced oxidative stress in rats of Ex group. However, SCE enhanced the fatigue-induced upregulation of Nrf2 and expression of HO-1, Trx, GCLC, and GCLM, which were downstream antioxidative genes product of the Nrf2/ARE pathway (Figures 3(b) and 3(c)). Thus, modulation of Nrf2/ARE signal pathway is likely to play a critical role in prevention of the muscle fatigue from the oxidative damage by SCE.

## 4. Conclusions

This study showed that SCE prepared by a superfine grinding technology has not only* in vitro* antioxidant activities but also* in vivo* antifatigue effects in rats. SCE has an ultrafine particle size and high content of antioxidants. The* in vivo* experiments study revealed that rats supplemented with SCE exhibited an increase in exhaustive swimming time. Meanwhile, improved exercise metabolism and activated energy metabolic reactions as evidenced by the increasing levels of blood glucose and of liver and muscle glycogen were observed in rats supplemented with SCE. Furthermore, rats supplemented with SCE showed a reduction of the accumulated by-products of metabolism, an inhibition of exercise-induced lipid peroxidation, and an improvement of the endogenous cellular antioxidant capacity by increasing the activities of antioxidant enzymes and upregulating Nrf2/ARE mediated antioxidant enzymes expression. Therefore, SCE supplementation can increase endurance capacity and facilitate recovery from fatigue in rats. The results provide an important basis for developing SCE as a novel antioxidant and antifatigue compound.

## Supplementary Material

The main antioxidants contents of SCE, such as oleanolic acid, ursolic acid and the presentative phenolics including gallic acid, ellagic acid, chlorogenic acid, caffeic acid, catechin and quercetin were measured with HPLC method. The related materials and methods were demonstrated as the following.

## Figures and Tables

**Table 1:** Primers for real-time PCR assay.

Gene	Primer (5′-3′)
HO-1	F: ATGACACCAAGGACCAGAGC R: GTAAGGACCCATCGGAGAAGC

Trx	F: CTGCTTTTCAGGAAGCCTTG R: TGTTGGCATGCATTTGACTT

GCLM	F: ACTGACTTAGGAGCATAACTTACC R: AAGAATATCTGCCTCAATGACACC

GCLC	F: AAGCCATTCACTCCAGATTTTACC R: ACAACAAACTTCAACGCAAAGC

*β*-actin	F: GCCGCCAG CTCACCATGGATG R: GACCCCGTCACCGGAGTC CA

**Table 2:** The main antioxidants contents and *in vitro* antioxidant activity of SCE.

	SCE	TCE
Oleanolic acid (%)	0.326 ± 0.064^*^	0.208 ± 0.031
Ursolic acid (%)	0.189 ± 0.031^*^	0.143 ± 0.015
Total flavones (mg/g)	49.15 ± 4.18^*^	25.24 ± 2.63
Gallic acid (mg/g)	6.80 ± 0.32^*^	4.38 ± 0.22
Ellagic acid (mg/g)	9.42 ± 1.45^*^	6.42 ± 1.73
Chlorogenic acid (mg/g)	2.01 ± 0.38^*^	1.24 ± 0.26
Caffeic acid (mg/g)	1.57 ± 0.31^*^	0.34 ± 0.26
Catechin (mg/g)	1.95 ± 0.65^*^	0.75 ± 0.23
Quercetin (mg/g)	11.28 ± 2.39^*^	7.03 ± 1.51
Total saponins (mg/g)	18.105 ± 0.623^*^	11.214 ± 0.409
Ascorbic acid (mg/g)	3.52 ± 0.83^*^	0.09 ± 0.02
Superoxide dismutase activity (U/mg)	68.895 ± 5.35^*^	3.125 ± 1.23
SCD^a^ (mg DPPH/g)	4.15 ± 1.81^*^	1.28 ± 0.6
SC-SAR^b^ (U/g)	2207 ± 163^*^	848 ± 94
FRAP^c^ (mmol Fe^2+^/g)	0.62 ± 0.03^*^	0.24 ± 0.03

Values represent means ± SD; *n* = 3;^*^ *P* < 0.05 versus TCE group; ^a^SC-DPPH, scavenging capacity of DPPH·(1,1-diphenyl-2-picrylhydrazyl free radical); ^b^SC-SAR, scavenging capacity of superoxide anion radical; ^c^FRAP, ferric reducing antioxidant power.

## Abbreviations

ARE: Antioxidant response element
BUN: Blood urea nitrogen
CAT: Catalase
GSH-Px: Glutathione peroxidase
LA: Lactic acid
TAOC: Total antioxidative capacity
TPC: Total flavones content
MDA: Malondialdehyde
SC-DPPH: 1,1-Diphenyl-2-picrylhydrazyl scavenging capacity
FARP: Ferric reducing antioxidant power
PBS: Phosphate buffer solution
SC-SAR: Superoxide anion radical scavenging capacity
SOD: Superoxide dismutase
Nrf2: Nuclear factor erythroid 2-related factor 2
HO-1: Heme oxygenase-1
Trx: Thioredoxins
GCLC: Glutamate-cysteine ligase catalytic subunit
GCLM: Glutamate-cysteine ligase modifier subunit.
