# Supplementary material for: Supplementation of Superfine Powder Prepared from Chaenomeles speciosa Fruit Increases Endurance Capacity in Rats via Antioxidant and Nrf2/ARE Signaling Pathway
Source: Evid Based Complement Alternat Med. 2014 Dec 24;2014:976438. doi: 10.1155/2014/976438 (PMC4290570; doi:10.1155/2014/976438)
Supplement: Supplementary file 1 — The main antioxidants contents of SCE, such as oleanolic acid, ursolic acid and the presentative phenolics including gallic acid, ellagic acid, chlorogenic acid, caffeic acid, catechin and quercetin were measured with HPLC method. The related materials and methods were demonstrated as the following. [file 976438.f1.doc]

**Supplementary materials and methods**

***1. Oleanolic acid and Ursolic acid assay with HPLC***

HPLC analysis of OA and UA was carried out on an Alliance HPLC system (Waters, USA), equipped with a venusil ABS-C_18_ column (4.6 mm× 250mm × 5μm). The powder (1.0 g) of SCE or TCE was soaked in 50ml ethanol for 30 min, and after ultrasonic extraction (40 kHz, 5 min), the samples were vacuum-concentrated and metered volume with 95% ethanol to 10ml before injection. The mobile phase consisted of methyl cyanides and 1.5% acetic acid in water (85:15, v/v) with a solvent flow rate of 0.8 ml/min, and the column temperature was kept at 30℃. The effluent was measured at a wavelength of 195 nm for the detection of OA and UA.

***2. Determination of*** ***representative phenolics by HPLC***

HPLC analysis of the representative phenolics was carried out on the Alliance HPLC system, equipped with a Zorbax SB-Aq C18 (250 mm×4.6 mm × 5μm) column. The powder (1.0 g) of SCE or TCE was soaked in 100ml ethanol solution at 50% (v/v) for 12 h at room temperature. Each sample was extracted three times with 25 ml each, for 30 min in reflux. The three parts extracted by reflux were mixed and the obtained samples were submitted to HPLC analysis. The mobile phase consisted of methyl cyanides and methanol in water (60:40, v/v) with a solvent flow rate of 0.8 ml/min, and the column temperature was kept at 30℃. The effluent was measured at 325 and 370nm for the detection of gallic acid, ellagic acid, chlorogenic acid, caffeic acid, catechin and quercetin.
